# Effects of Long-Term Organic Fertilization on Productivity, Stability, and Nitrogen Use Efficiency in Rotation Systems of the Hetao Irrigation District

**DOI:** 10.3390/plants15091400

**Published:** 2026-05-03

**Authors:** Xue Zhang, Lanfang Bai, Na Zhao, Yongqiang Wang, Yu Yao, Fugui Wang, Zhen Wang, Hongwei Liang, Xiaohong Li, Jufeng Cao, Zhigang Wang

**Affiliations:** 1 College of Agronomy, Inner Mongolia Agricultural University, Hohhot 010019, China; xzhang1@emails.imau.edu.cn (X.Z.); wangyongqiang@nwafu.edu.cn (Y.W.); 15698668386@163.com (Y.Y.); fgwang2008@163.com (F.W.); cau1022@imau.edu.cn (Z.W.); 2 Inner Mongolia Engineering Research Center for Intelligent Water-Fertilizer Management Technology and Equipment, Hohhot 010019, China; 3 Bio-Agriculture Institute of Shaanxi, Xi’an 710043, China; zhaona@xab.ac.cn; 4 Inner Mongolia Academy of Agricultural and Animal Husbandry Sciences, Hohhot 010031, China; hwliang@emails.imau.edu.cn; 5 Bayannur Academy of Agricultural and Animal Sciences, Linhe, Bayannur 015400, China; 13789482255@163.com (X.L.); caojufeng1978@163.com (J.C.)

**Keywords:** crop rotation system, organic fertilization, nitrogen use efficiency, yield stability, crop yield

## Abstract

This study investigated how different organic fertilization practices affect productivity, stability, and nitrogen use efficiency in the rotation systems of the Hetao Irrigation District. The research was based on a long-term field experiment (2015–2025), with a chemical fertilizer-only treatment as the control (CK). Four organic fertilization treatments were evaluated: farmyard manure application (CM), straw incorporation (CS), green manure cultivation and incorporation (CG), and a combined green manure plus straw treatment (CGS). Based on three consecutive years of observations (2023–2025), the impacts of these treatments on crop yield, yield composition and stability, plant nitrogen accumulation and allocation, and nitrogen use efficiency were systematically analyzed. Both CM and CS significantly increased maize equivalent yield (MEY) compared with the other treatments, by 33.68–66.04% and 16.05–24.21%, respectively. CM’s productivity advantage was primarily driven by higher biomass accumulation, whereas CS’s advantage was largely due to improvements in the harvest index. In terms of stability, CM exhibited the lowest coefficient of variation (CV), indicating the highest static stability, while CS showed a regression coefficient (*b_i_*) close to 1, indicating stronger dynamic stability. CM also significantly enhanced total plant nitrogen accumulation, nitrogen recovery efficiency (NRE), and nitrogen use efficiency (NUE), while optimizing nitrogen allocation to grain. CS significantly improved nitrogen internal efficiency (NIE), promoting more efficient conversion of absorbed nitrogen into grain yield. CG and CGS did not show clear advantages across productivity, stability, or most nitrogen use efficiency-related indices. Overall, in the Hetao Irrigation District, farmyard manure application is an effective strategy for achieving both high and stable yields, whereas straw incorporation offers stronger environmental adaptability. Both practices represent practical and effective approaches for improving the sustainability of rotation systems.

## 1. Introduction

The Hetao Irrigation District in northern China is a typical semi-arid irrigated agricultural region, primarily cultivating sunflower, wheat, and maize. It plays a critical role in sustaining agricultural productivity and ensuring national food security [1]. In this region, low annual precipitation and high evapotranspiration make crop production heavily dependent on irrigation-driven water and nutrient inputs [2,3]. Long-term reliance on high chemical fertilizer application, particularly nitrogen, has maintained yields but has also led to low nitrogen use efficiency, declining soil fertility, and diminishing marginal returns on nitrogen application [4,5,6]. Meanwhile, market-driven cultivation of high-value crops such as sunflower often involves repeated monoculture, which exacerbates continuous cropping obstacles and limits both yield stability and overall productivity [7,8]. Global meta-analyses and systematic reviews indicate that diversified crop rotations not only mitigate yield losses caused by continuous cropping but also enhance yield stability, improve nutrient use efficiency, and increase economic returns at the rotation system level [9,10]. Therefore, continuous soil fertility improvement and optimized nitrogen management are essential for enhancing productivity and sustainability in semi-arid irrigated agricultural systems.

Organic fertilization is an important approach to improving soil quality, regulating nitrogen source composition, and synchronizing nitrogen supply with crop demand [11]. In irrigated agriculture, it is widely applied to promote yield formation and enhance nitrogen use efficiency [12,13]. Previous studies have shown that the application of farmyard manure or combined organic-inorganic fertilization can increase soil organic matter, thereby enhancing plant nitrogen uptake and accumulation and ultimately improving yield. Straw incorporation can regulate nitrogen mineralization and immobilization processes, improving the synchronization between nitrogen supply and crop demand and enhancing nitrogen use efficiency [14,15,16]. Legume-based green manures contribute nitrogen inputs through biological nitrogen fixation, thereby improving nitrogen supply for subsequent crops [17,18,19]. In addition to their effects on yield and nitrogen use efficiency, organic fertilization practices have increasingly been recognized for their role in regulating the stability of agricultural systems. Continuous inputs of organic materials can improve soil structure, enhance soil nutrient buffering capacity, and strengthen system resilience to environmental fluctuations, thereby maintaining yield stability [20,21,22]. However, due to differences in nutrient release patterns among organic materials and their synchronization with crop nutrient demand [23], their effects on system productivity stability are not consistent [24]. Overall, existing studies have mainly focused on the roles of organic fertilization in improving yield, nitrogen use efficiency, or soil fertility, whereas systematic comparisons of the effects of long-term organic fertilization on system productivity stability and nitrogen use efficiency in rotation systems remain limited.

Building on this background, this study was based on a long-term field experiment (2015–2025) in the Hetao Irrigation District, focusing on a sunflower–wheat–maize rotation system. On the basis of conventional fertilization, four organic fertilization treatments were established: farmyard manure application (CM), straw incorporation (CS), green manure cultivation and incorporation (CG), and a combined treatment of green manure incorporation with straw return (CGS), with chemical fertilization alone serving as the control (CK). This study aimed to clarify how different organic fertilization practices influence system productivity and yield stability at the rotation scale through changes in yield formation components, and to further analyze their effects on nitrogen use efficiency based on plant nitrogen accumulation and allocation. On this basis, a comprehensive evaluation was conducted to compare the overall performance of different fertilization strategies, thereby providing a theoretical basis for optimizing organic fertilization strategies and achieving the coordinated improvement of productivity, stability, and resource use efficiency in irrigated agricultural systems.

## 2. Results

### 2.1. Effects of Organic Fertilization on System Productivity

**Figure 1:**
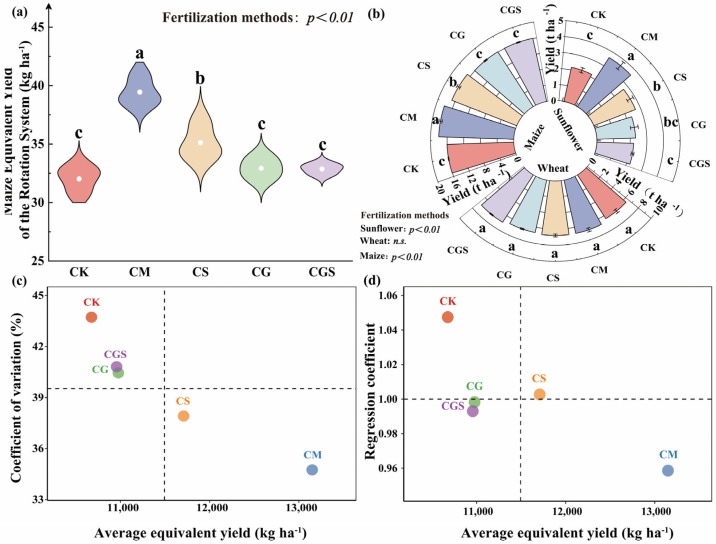
Effects of different organic fertilization treatments on rotation system productivity and yield stability. (**a**) Rotation system productivity under different fertilization treatments, expressed as maize equivalent yield (MEY). Error bars represent standard deviations (SD). (**b**) Yields of sunflower, wheat, and maize under each fertilization treatment. (**c**) Static stability of rotation system productivity, evaluated using the coefficient of variation (CV) of MEY. Each point represents one fertilization treatment. The horizontal dashed line indicates the overall mean MEY across treatments, and the vertical dashed line indicates the mean CV. Lower CV values indicate greater static stability. (**d**) Dynamic stability of rotation system productivity, assessed using the Finlay–Wilkinson regression coefficient (*b_i_*). The horizontal dashed line (*b_i_* = 1) represents a neutral response to environmental change, and the vertical dashed line indicates the overall mean MEY across treatments. Values of *b_i_* < 1 indicate lower environmental sensitivity and stronger buffering capacity, whereas *b_i_* > 1 indicate greater responsiveness under favorable environmental conditions. Different letters indicate significant differences among treatments (*p* < 0.05), determined by analysis of variance (ANOVA).

Organic fertilization practices had a highly significant effect on maize equivalent yield (MEY) across the rotation system (Figure 1a; *p* < 0.01). Both CM and CS significantly increased total MEY by 19.78–23.16% and 6.67–9.67%, respectively, compared with the other treatments. Among all treatments, CM achieved the highest MEY, which was significantly higher than that of CS by 12.29%. No significant differences were observed among CG, CGS, and CK. Further analysis revealed that the differences in MEY among treatments were primarily attributed to the sunflower and maize seasons, whereas no significant differences were observed during the wheat season (Figure 1b). In the sunflower season, MEY under CM and CS was significantly increased by 55.13–66.04% and 16.05–24.21%, respectively (*p* < 0.01). In the maize season, the increases were comparatively smaller, at 12.05–12.39% for CM and 4.65–4.96% for CS (*p* < 0.01).

Comparison of MEY across treatments showed that CM and CS were consistently above the overall mean, whereas CG, CGS, and CK were below the average (Figure 1c,d). In terms of static stability, the coefficient of variation (CV) ranked as CM < CS < CGS < CG < CK (Figure 1c), indicating that CM and CS exhibited higher stability with lower interannual variability. Dynamic stability analysis (Figure 1d) showed that the regression coefficients (*b_i_*) of CS and CG were closest to 1, followed by CGS, whereas CM deviated more from 1. This indicates that CS and CG had stronger adaptability to environmental variation and higher dynamic stability, while CM was more sensitive to environmental changes.

### 2.2. Effects on Biomass and Harvest Index

**Figure 2:**
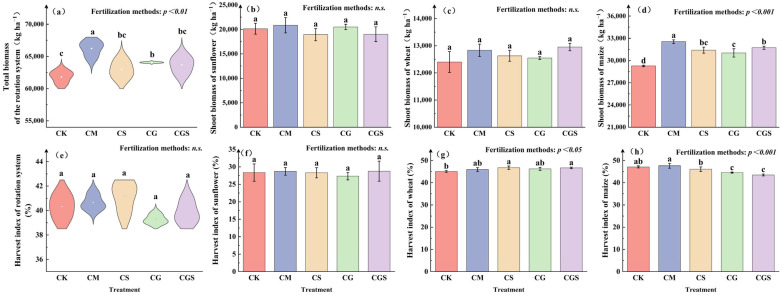
Effects of different organic amendment treatments on biomass and harvest index in the rotation system. (**a**) Biomass of the sunflower–wheat–maize rotation system; (**b**) Biomass of sunflower; (**c**) Biomass of wheat; (**d**) Biomass of maize. Dots within the violin plots represent mean values. (**e**) Harvest index of the sunflower–wheat–maize rotation system; (**f**) Harvest index of sunflower; (**g**) Harvest index of wheat; (**h**) Harvest index of maize. Error bars represent standard deviations. Different letters indicate significant differences among treatments (*p* < 0.05), determined by analysis of variance (ANOVA).

Organic fertilization had a highly significant effect on total biomass in the rotation system (Figure 2a; *p* < 0.01). Among the treatments, CM produced the highest total biomass (66,228.61 kg ha^−1^), which was significantly higher than that of the other treatments by 3.37–7.15%. Further analysis indicated that the increase in total biomass was mainly driven by the maize season. In the maize season (Figure 2d), aboveground biomass under all fertilization treatments was significantly higher than that of CK (*p* < 0.001), following the order CM > CGS > CS > CG > CK. Biomass under CM reached 32,546.62 kg ha^−1^, which was significantly higher than that of the other treatments by 2.6–11.18%. In contrast, aboveground biomass did not differ significantly among treatments during the wheat and sunflower seasons (Figure 2b,c; *p* > 0.05).

At the system level, organic fertilization had no significant effect on the harvest index (Figure 2e; *p* > 0.05). However, significant differences were observed at the seasonal scale (Figure 2f–h). In the wheat season (Figure 2g), harvest index values under the organic fertilization treatments were 2.03–3.83% higher than those of CK, with CS showing the largest numerical increase. In the maize season (Figure 2h), CM had the highest harvest index, although it did not differ significantly from CK.

### 2.3. Effects of Organic Fertilization on Nitrogen Use Efficiency

**Figure 3:**
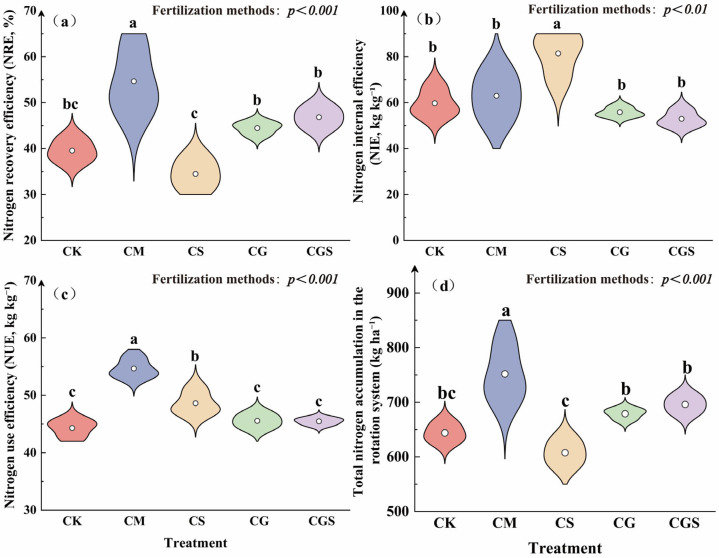
Effects of different organic amendment practices on nitrogen use efficiency and total nitrogen accumulation in the rotation system. (**a**–**c**) Nitrogen recovery efficiency (NRE) (**a**), nitrogen internal efficiency (NIE) (**b**), and nitrogen use efficiency (NUE) (**c**) in the sunflower–wheat–maize rotation system under different organic amendment practices; (**d**) total nitrogen accumulation in the sunflower–wheat–maize rotation system under different organic amendment practices. Dots within the violin plots represent the mean values. Error bars represent standard deviations. Different letters indicate significant differences among treatments (*p* < 0.05), determined by analysis of variance (ANOVA). Different letters indicate significant differences among treatments (*p* < 0.05), determined by analysis of variance (ANOVA).

Organic fertilization strategies had significant effects on nitrogen use efficiency in the rotation system (Figure 3a–c). Among the treatments, CM exhibited the highest nitrogen recovery efficiency (NRE), which was significantly higher than that of the other treatments by 16.66–58.47% (Figure 3a). CS showed the highest nitrogen internal efficiency (NIE), reaching 81.39 kg kg^−1^, and it was significantly higher than that of all other treatments (Figure 3b; *p* < 0.01). In terms of nitrogen use efficiency (NUE), both CM and CS were significantly higher than the other treatments, with increases of 20.00–23.42% and 6.74–9.78%, respectively (Figure 3c; *p* < 0.001). In contrast, CG and CGS did not differ significantly from CK in NRE, NIE, or NUE.

### 2.4. Effects of Organic Fertilization on Plant Nitrogen Accumulation

**Figure 4:**
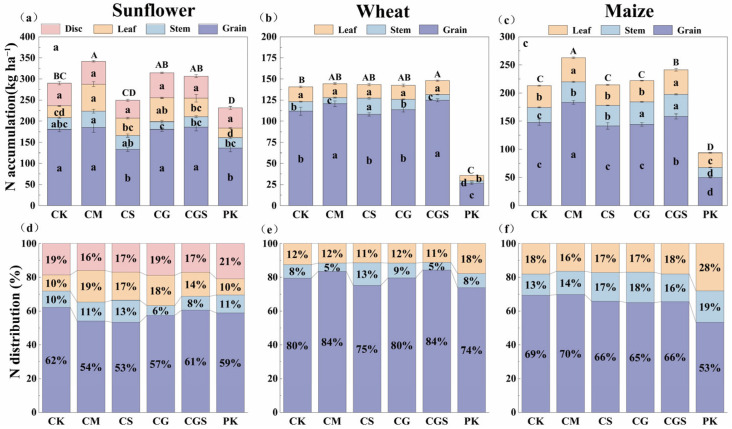
Effects of different organic amendment practices on plant nitrogen accumulation. (**a**–**d**) Biomass of sunflower (**a**), wheat (**b**), and maize (**c**) under different organic amendment treatments; (**e**–**h**) harvest index of sunflower (**d**), wheat (**e**), and maize (**f**) under different organic amendment treatments. Different lowercase letters within the bars indicate significant differences among organs under different treatments at the 5% significance level, while different uppercase letters above the bars indicate significant differences in aboveground plant nitrogen accumulation among different treatments at the 5% significance level. Error bars represent standard deviations. Significance was determined by analysis of variance (ANOVA).

Organic fertilization significantly affected total plant nitrogen accumulation in the rotation system (Figure 3d). CM consistently showed the highest nitrogen accumulation in the sunflower, wheat, and maize seasons (Figure 4a–c). The total plant nitrogen accumulation under CM reached 751.71 kg ha^−1^, which was significantly higher than that of the other treatments by 8.02–23.72% (Figure 3d). Analysis of nitrogen allocation among plant organs (Figure 4d–f) revealed clear differences among treatments. In the sunflower season, nitrogen under CM was mainly allocated to grains and leaves, whereas in other treatments it was primarily distributed between grains and capitula. Notably, leaf nitrogen accumulation under CM was 2.31 times that of CK. In the wheat season, nitrogen was predominantly allocated to grains across all treatments, with CGS and CM showing the highest proportions (both 84%), while stems accounted for approximately 5%. In the maize season, nitrogen was mainly distributed between grains and leaves, and CM showed the highest proportion of nitrogen allocated to grains, reaching 69% of total plant nitrogen accumulation.

### 2.5. Comprehensive Performance of Fertilization Strategies

**Figure 5:**
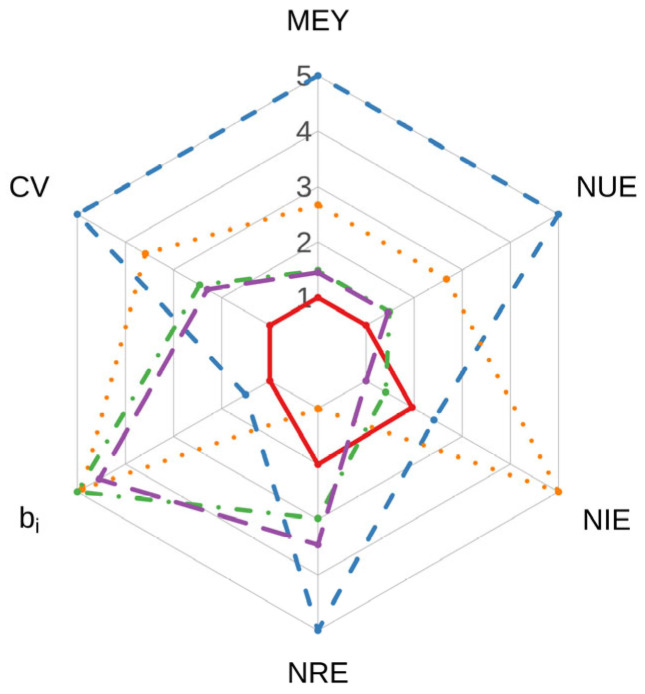
Comprehensive scores of different organic fertilization treatments, calculated based on standardized values of maize equivalent yield (MEY), coefficient of variation (CV), regression coefficient (*b_i_*), nitrogen recovery efficiency (NRE), nitrogen internal efficiency (NPE), and nitrogen use efficiency (NUE). Red, CK; blue, CM; orange, CS; green, CG; purple, CGS.

The comprehensive index (CI) of different organic fertilization treatments followed the order: CM > CS > CG > CGS > CK (Figure 5; Table 1). CM achieved the highest CI value (3.99), mainly due to its superior performance in productivity and nitrogen-related indicators (MEY, NRE, and NUE), as well as its high score in static stability (CV). CS ranked second (3.30), primarily because it had the highest NIE and consistently high values for MEY, NUE, and stability-related indicators (*b_i_* and CV). CG ranked third (2.47), largely due to its highest *b_i_* value, indicating stronger adaptability to environmental variation.

## 3. Discussion

### 3.1. Effects of Organic Fertilization on Productivity and Stability in the Rotation System

Organic fertilization strategies regulate yield components in a differential manner, thereby driving yield formation and leading to synergy or trade-offs between productivity and stability at the rotation scale. In this study, the pathways of yield improvement differed markedly among treatments, with CM and CS showing the strongest productivity performance. The high yield under CM was primarily attributed to a significant increase in total biomass, whereas the yield advantage of CS was mainly driven by an improved harvest index. Previous studies provide strong support for these differentiated pathways. Shi et al. reported [25], based on a meta-analysis of 537 global experiments, that organic fertilization increased aboveground biomass by 56%, which was significantly higher than the 42% increase under inorganic fertilization, indicating that the yield-enhancing effect of organic fertilization is primarily achieved through promoting biomass accumulation. Long et al. [26], based on a 14–15-year long-term field experiment, found that CS optimized source–sink balance by promoting nitrogen accumulation, increasing the grain-to-leaf ratio, and improving harvest index, ultimately enhancing crop yield. Cheng et al. [27], from a six-year, long-term trial, demonstrated that high-input straw pellet return facilitated the translocation of dry matter to grains, increasing conversion efficiency and grain yield. These studies indicate that the yield-enhancing effects of organic amendments can occur through two pathways: increasing biomass or optimizing the allocation of assimilates, which aligns with the different yield formation mechanisms observed in this study. Consequently, CM and CS represent two distinct yield formation strategies. In contrast, CG and CGS did not show substantial productivity advantages under the conditions of this study. However, this does not imply that green manure lacks yield potential. Huang et al. [18], based on a global meta-analysis, reported that green manure incorporation increased crop yield by 3.71% and nitrogen use efficiency by 24.41%, with leguminous green manures performing best in low-fertility soils under warm and humid climates. Liang et al. [28], from a China-based meta-analysis, found that leguminous green manure incorporation increased yields of the three major cereals by 12.60%, with rice, maize, and wheat showing respective gains of 19.22%, 16.70%, and 9.49%, particularly under sufficient precipitation, favorable temperatures, and low-organic-matter soils. The limited contribution of green manure in this study is likely due to its early harvest at full flowering, which restricts growth duration and biomass accumulation, limiting its sustained contribution to productivity over the rotation cycle. Therefore, although green manure retains inherent yield potential, its contribution to rotation system productivity is lower than that of CM or CS treatments.

Further analysis of yield stability revealed distinct stability characteristics among different organic fertilization strategies within the rotation system. The CM treatment exhibited the highest static stability (lowest CV), indicating the smallest interannual yield variation. However, its regression coefficient deviated substantially from 1, suggesting weaker dynamic stability and greater sensitivity to environmental variability. A global meta-analysis by Young et al. [29] similarly demonstrated that the yield benefits of organic fertilization are highly dependent on environmental conditions and vary across different climatic and soil contexts. The coexistence of low interannual yield variability and high environmental sensitivity reflects a potential trade-off between yield potential and environmental adaptability in crop production systems [24]. This characteristic is likely related to the biomass-driven yield formation pathway under CM. High yields in the CM treatment primarily rely on substantial biomass accumulation, which is typically associated with greater resource demand. Luo et al. [30], based on a global meta-analysis, reported that farmyard manure achieves the greatest yield increases among organic amendments; however, this effect depends on sufficient nutrient supply and is characterized by a high-input, high-output pattern, which is likely an important reason for the weaker dynamic stability of the CM treatment. In contrast, although the CS treatment produced slightly lower yields, its regression coefficient was closer to 1, indicating better dynamic stability and stronger adaptability across varying environmental conditions. This difference is primarily attributable to the distinct yield formation pathways of the two treatments. A meta-analysis by He et al. [31]. showed that long-term straw return can increase average crop yield by 12% while also enhancing yield stability. Similarly, Indoshi et al. [32]. demonstrated in semi-arid regions of East Africa that straw return improves assimilate partitioning to grain, allowing crops to maintain relatively high yields even in years with low rainfall and high temperatures, thereby exhibiting lower sensitivity to environmental fluctuations and greater interannual stability. Overall, CM and CS represent two distinct stability pathways within the rotation system. CM, driven by biomass accumulation, achieves higher static stability, whereas CS, through improved assimilate partitioning efficiency, exhibits superior dynamic stability. These findings suggest that management of semi-arid irrigated rotation systems should strike a balance between productivity enhancement and risk buffering capacity. Focusing solely on maximizing yield or minimizing variability may overlook the vulnerability of the system under extreme climatic conditions.

### 3.2. Effects of Organic Fertilization on Crop Nitrogen Accumulation and the Formation of Nitrogen Use Efficiency

Organic fertilization regulates crop nitrogen uptake and its partitioning among plant organs, thereby driving the coordinated improvement of yield and nitrogen use efficiency at the rotation system scale. In this study, within the sunflower–wheat–maize rotation system, the CM treatment showed clear advantages in plant nitrogen accumulation, nitrogen recovery efficiency (NRE), and nitrogen use efficiency (NUE). Kamiji et al. [33] reported that under low nitrogen supply, aboveground biomass is the primary driver of nitrogen uptake in wheat. Similarly, Duncan et al. [34] indicated that improvements in nitrogen use efficiency are often accompanied by increases in biomass and nitrogen uptake. In addition, van der Sloot et al. [35] demonstrated that the carbon-to-nitrogen ratio of organic materials is a key factor regulating crop growth. Therefore, the superior nitrogen use efficiency under CM may be associated with its moderate C:N ratio and higher biomass production. Previous studies further indicate that nitrogen allocation patterns within the plant are also critical for determining nitrogen use efficiency [36,37,38]. In this study, grain served as the primary sink organ for nitrogen accumulation, followed by leaves, while stems contributed relatively little. Pask et al. [39] reported that leaves accounted for 36–42% of aboveground nitrogen accumulation at anthesis, highlighting their role as a major nitrogen reservoir. Yin et al. [40] further demonstrated that grain nitrogen is largely derived from the remobilization of nitrogen from vegetative organs such as leaves, and that higher leaf nitrogen reserves help sustain photosynthetic activity during grain filling. These findings collectively support a nitrogen allocation pattern in which leaves function as the main storage pool and grains as the final sink. Accordingly, the CM treatment enhanced nitrogen use efficiency at the rotation scale by optimizing nitrogen partitioning between leaves and grains, increasing leaf nitrogen storage, and improving the efficiency of nitrogen remobilization to grains. In contrast, the advantage of the CS treatment in nitrogen use efficiency was mainly reflected in nitrogen internal efficiency (NIE). Ciampitti and Prasad [41] emphasized that the proportion of nitrogen allocated to grains is a key determinant of nitrogen use efficiency. Tian et al. [42] showed that straw return promotes nitrogen translocation to grains. In this study, the higher harvest index observed under CS is consistent with this mechanism, indicating that its nitrogen efficiency advantage primarily arises from more efficient redistribution of absorbed nitrogen. By comparison, the CG and CGS treatments did not exhibit clear advantages in nitrogen use efficiency indices. This may be related to the relatively limited biomass accumulation of green manure, which restricts the development of sufficient aboveground N uptake capacity and thereby constrains overall crop nitrogen acquisition. A meta-analysis by Xu et al. [43] showed that although green manure can enhance nitrogen uptake and nitrogen use efficiency of subsequent crops, the proportion of nitrogen directly utilized by crops remains relatively low, indicating uncertainty and environmental dependence in its contribution to nitrogen supply. However, Huang et al. [18] reported that leguminous green manure achieves the greatest yield benefits under low soil fertility and warm, humid conditions. Therefore, under semi-arid irrigated conditions, the potential of green manure to enhance nitrogen use efficiency may not be fully realized. In summary, different organic fertilization strategies regulate nitrogen use efficiency through distinct pathways. The CM treatment enhances NRE and NUE primarily by strengthening nitrogen uptake capacity based on high biomass accumulation, whereas the CS treatment improves NIE by optimizing nitrogen allocation and translocation to grains. These differentiated mechanisms jointly underpin the coordinated enhancement of productivity and resource use efficiency in the rotation system.

## 4. Materials and Methods

### 4.1. Site Description

This study was based on a long-term organic fertilization field experiment (2015–2025) in the Hetao Irrigation District. Field monitoring from 2023 to 2025 was conducted at the Yuanzhiqu Experimental Station of the Bayannur Academy of Agricultural and Animal Husbandry Sciences, Inner Mongolia, China (40°90′ N, 107°17′ E). Maize was the preceding crop for the 2023 growing season. Soil particle size distribution was determined using a laser particle size analyzer (Mastersizer 3000, Malvern Panalytical Ltd., Malvern, UK), and soil texture was classified according to the USDA soil texture classification system [44]. The soil at the experimental site was classified as loam. The experimental site is located in the central Hetao Irrigation District at an elevation of 1035 m and is characterized by a temperate continental monsoon climate. The mean annual temperature is 6.8 °C, with mean annual precipitation of 168 mm and annual evaporation of 2030–3180 mm. The frost-free period is approximately 135–145 days. The initial soil physicochemical properties (0–20 cm) are presented in Table 2.

### 4.2. Experimental Design and Field Management

**Figure 6:**
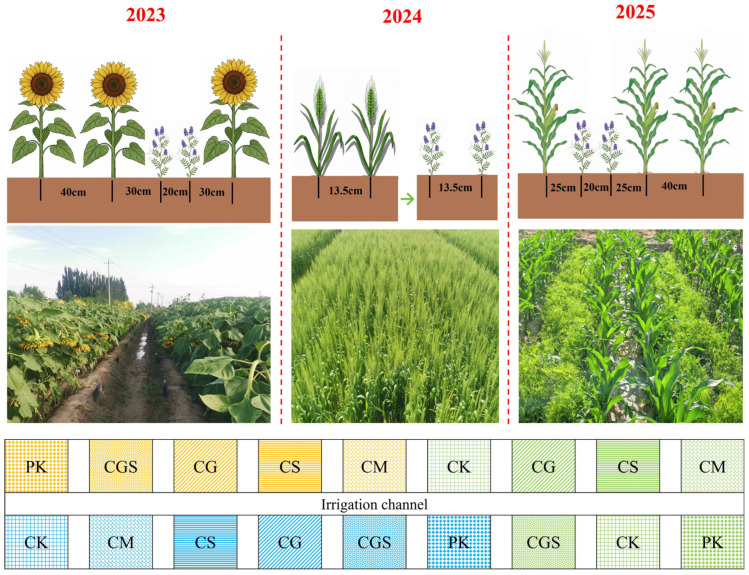
Schematic diagram and field layout of the sunflower–wheat–maize rotation system.

The long-term organic fertilization experiment (2015–2025) in the Hetao Irrigation District followed a three-year wheat–maize–sunflower rotation system (wheat in 2015, maize in 2016, sunflower in 2017, and repeating thereafter). The experiment was arranged in a randomized complete block design with six treatments, including four organic fertilization treatments: farmyard manure application (CM), straw incorporation (CS), green manure cultivation and incorporation (CG), and a combined treatment of green manure incorporation with straw return (CGS). Chemical fertilization alone served as the control (CK). In addition, a nitrogen-omission treatment (PK), in which only phosphorus and potassium fertilizers were applied without nitrogen or organic inputs, was included for the calculation of nitrogen use efficiency-related indices. Each treatment had three replicates, giving a total of 18 plots, with an area of 40 m^2^ (8 m × 5 m) per plot. The layout of the experimental plots is shown in Figure 6. All treatments received identical rates of chemical fertilizers, with detailed application rates provided in Table 3. Nitrogen, phosphorus, and potassium fertilizers were applied as urea (Sinochem Group, Beijing, China), diammonium phosphate (Yuntianhua Group, Yunnan, China), and potassium chloride (Qinghai Salt Lake Industry Co., Ltd., Qinghai, China), respectively, while triple superphosphate was used as the phosphorus source in the PK treatment. Nitrogen fertilizer was applied in split doses, with 30% as a basal application and 70% as topdressing. All phosphorus fertilizer was applied as basal fertilizer, while potassium fertilizer was applied at 70% as basal and 30% as topdressing. Topdressing was applied with irrigation at key growth stages: the budding stage of sunflower, the jointing stage of wheat, and the large bell mouth stage of maize.

The organic fertilization treatments were implemented as follows. In the farmyard manure treatment (CM), well-composted sheep manure was selected as the organic fertilizer source because it is widely available and commonly used in the local agro-pastoral system of the Hetao Irrigation District. Moreover, its relatively stable properties after composting and reliable long-term availability make it suitable for long-term field experiments. The application rate was 7500 kg ha^−1^. In the straw incorporation treatment (CS), all crop residues were chopped and fully returned to the soil after each harvest. For the green manure treatment (CG), hairy vetch (*Vicia villosa*, Turkmen cultivar) was used. It was sown between wide rows during the sunflower and maize seasons at seeding rates of 75 and 45 kg ha^−1^, respectively, and grown as a subsequent crop during the wheat season at a seeding rate of 45 kg ha^−1^. In the combined treatment (CGS), straw incorporation and green manure cultivation with subsequent incorporation were implemented simultaneously. All organic materials were incorporated into the soil by plowing after harvest. The nutrient contents of the organic materials are provided in Table 4.

The crop varieties and planting densities were as follows: sunflower (SH363) at 33,000 plants ha^−1^, wheat (Yongliang No. 4) at a seeding rate of 450 kg ha^−1^, and maize (Ximeng 568) at 75,000 plants ha^−1^. Irrigation was conducted using traditional border irrigation, with timing and amounts determined according to local practices and Yellow River water allocation schedules [4,45,46]. All other field management practices were consistent with local conventional practices.

### 4.3. Biomass

At crop maturity, three uniform plants were sampled per plot for sunflower and maize. Sunflower plants were separated into head, stem, leaves, and grain, while maize plants were divided into stem, leaves, and grain; each component was placed into mesh bags. For wheat, a 1 m^2^ area per plot was sampled and separated into stems, leaves, and grains in mesh bags. All samples were initially heated at 105 °C for 30 min to inactivate enzymes, and then oven-dried at 80 °C until constant weight (DHG-9070A, Shanghai Yiheng Scientific Instrument Co., Ltd., Shanghai, China). The biomass of each organ was recorded and subsequently converted to per-area biomass (kg ha^−1^).

### 4.4. Plant Nitrogen Content

Dried plant samples at maturity were ground separately by organ and thoroughly homogenized. Total nitrogen content of each organ was determined using the Kjeldahl method (K1100, Hanon Instruments Co., Ltd., Jinan, China).

### 4.5. Yield and Plant Assessment

At crop maturity, sunflower heads, wheat spikes, and maize ears were counted in each plot for yield determination. Additionally, ten uniform plants per plot were randomly selected for detailed assessment, and grain moisture content was measured using a grain moisture meter (PM-8188-A, Kett Electric Laboratory, Tokyo, Japan). Green manure was harvested at full bloom across all plots. A 1 kg subsample was oven-dried to constant weight to determine biomass, which was then converted to per-area biomass (kg ha^−1^).

### 4.6. Data Calculations

The nitrogen accumulation in individual plant organs and the total aboveground nitrogen accumulation were calculated as follows:

(1) $$U_{i}=\frac{W_{i}\;\times \;C_{i}}{1000}$$

(2) $$U=\sum U_{i}$$

where *U_i_* is the nitrogen accumulation in organ i (kg ha^−1^), *W_i_* is the biomass of organ i (kg ha^−1^), and *C_i_* is the nitrogen concentration in organ i (%), with the factor 1000 used to adjust units. Total aboveground nitrogen accumulation (*U*, kg ha^−1^) is calculated as the sum of nitrogen accumulation across all plant organs.

To assess nitrogen use efficiency under different organic fertilization practices, the following indices were calculated.

(3) $$\mathrm{NRE}=\frac{U_{T}-U_{0}}{F_{N}}\times 100\%$$

(4) $$\mathrm{NIE}=\frac{Y_{T}-Y_{0}}{U_{T}-U_{0}}$$

(5) $$\mathrm{NUE}=\frac{Y_{T}-Y_{0}}{F_{N}}$$

Nitrogen recovery efficiency (NRE, %) reflects the proportion of applied nitrogen absorbed by the crop. Nitrogen internal efficiency (NIE, kg kg^−1^) represents the grain yield produced per unit of nitrogen absorbed by the plant, while nitrogen use efficiency (NUE, kg kg^−1^) represents the yield increase per unit of nitrogen applied. *U_T_* and *U*_0_ are the aboveground nitrogen accumulations in fertilized and unfertilized plots, respectively (kg ha^−1^), whereas *Y_T_* and *Y*_0_ are the grain yields in fertilized and unfertilized plots (kg ha^−1^). *F_N_* represents the amount of nitrogen applied (kg ha^−1^).

Harvest index (HI, %) is defined as the ratio of grain biomass to aboveground biomass, reflects the allocation of aboveground biomass to grain production, and was calculated as follows:

(6) $$\mathrm{HI}=\frac{W_{grain}}{W_{above}}\times 100\%$$

where *W_grain_* and *W_above_* are the grain biomass and aboveground biomass, respectively (kg ha^−1^).

To facilitate comparison of productivity across the rotation system, maize was selected as the reference crop because it is one of the dominant staple crops in the Hetao Irrigation District, with relatively stable yield and market price. All crop yields were standardized to maize equivalent yield (MEY, kg ha^−1^) [24].

(7) $$\mathrm{MEY}=\frac{P_{sunflower}}{P_{maize}}\times Y_{sunflower}+\frac{P_{wheat}}{P_{maize}}\times Y_{wheat}+Y_{maize}$$

During the study period (2015–2025), the average market prices for sunflower, wheat, and maize were 7.0, 3.0, and 2.3 Chinese yuan (CNY) per kg, respectively. In Equation (7), MEY represents maize equivalent yield (kg ha^−1^), *Y* is the crop yield (kg ha^−1^), *P* is the market price of the crop.

The stability of productivity under different organic fertilization practices was assessed using Maize Equivalent Yield (MEY) [24]. Dynamic stability was evaluated using the Finlay–Wilkinson regression coefficient (*b_i_*) [47]. For each treatment, MEY was regressed against an environmental index, defined as the mean MEY across all treatments in a given year, to represent that year’s environmental conditions. Interpretation of *bi* values is as follows: a *b_i_* close to 1 indicates broad adaptability to varying environmental conditions; *b_i_* < 1 indicates lower responsiveness to favorable environmental conditions, showing relatively stable performance in low-yield years; *b_i_* > 1 indicates stronger responsiveness to favorable conditions, resulting in higher potential yields in good years but greater yield risk in poor years. Static stability was measured by the coefficient of variation (CV), where lower CV values indicate smaller year-to-year fluctuations and higher stability. CV was calculated as:

(8) $$\mathrm{CV}=\frac{\mathrm{SD}}{\bar{x}}\times 100\%$$

where SD is the standard deviation of MEY across years for a treatment, and $\bar{x}$ is the mean MEY across years. CV reflects the year-to-year variability of system productivity, with lower CV values indicating smaller fluctuations and higher static stability.

To comprehensively evaluate the overall performance of different organic fertilization strategies within the rotation system, a composite index approach was adopted [48]. Six indicators were selected, including maize equivalent yield (MEY), coefficient of variation (CV), regression coefficient (*b_i_*), nitrogen recovery efficiency (NRE), nitrogen internal efficiency (NIE), and nitrogen use efficiency (NUE). To eliminate dimensional effects, all indicators were normalized to a scale ranging from 1 to 5 using range normalization. Indicators were classified as positive, negative, or target indicators. For positive indicators (MEY, NRE, NIE, NUE), higher values indicate better performance, and normalization was conducted as follows:

(9) $$S_{ij}=\frac{\left(5-1)\times (X_{ij}-X_{j,\;\min}\right)}{X_{j,\;\max}-X_{j,\;\min}}+1$$

where *X_ij_* and *S_ij_* represent the original and normalized values of the j-th indicator for the i-th treatment, respectively, and *X_j, max_* and *X_j, min_* denote the maximum and minimum values of that indicator across all treatments. This equation transforms the original values into a standardized scale ranging from 1 to 5, where higher values indicate better performance.

The regression coefficient (*b_i_*) was treated as a target indicator with an optimal value of 1. Therefore, its deviation from 1 was first calculated:

(10) $${X_{ij}}^{\prime }=\left|b_{i_{ij}}-1\right|$$

and then the deviation was incorporated into the normalization formula for negative indicators. For negative indicators (e.g., CV), lower values indicate better performance, and normalization was performed as:

(11) $$S_{ij}=\frac{4\times (X_{j,\;\max}-X_{ij})}{X_{j,\;\max}-X_{j,\;\min}}+1$$

where the factor 4 (i.e., 5 − 1) represents the length of the normalization interval, and adding 1 ensures that all normalized values fall within the range of 1–5. After normalization, all indicators were combined using an equal-weight method to calculate the comprehensive index (CI):

(12) $$\mathrm{CI}=\frac{1}{n}\;\sum_{j=1}^{n}\;S_{ij}$$

where CI ranges from 1 to 5, and *n* is the number of indicators (*n* = 6). A higher CI value indicates better overall system performance. The CI integrates multiple productivity, stability, and nitrogen use efficiency indicators into a single metric.

### 4.7. Data Analysis

All data were organized using Microsoft Excel 2021 (Microsoft Corp., Redmond, WA, USA) and analyzed with SPSS Statistics 27.0 (IBM Corp., Armonk, NY, USA). Differences among treatments were tested using the least significant difference (LSD) method. Dynamic stability was evaluated using Finlay–Wilkinson regression analysis in R software (version 4.4.3, R Foundation for Statistical Computing, Vienna, Austria), with regression coefficients (*b_i_*) calculated using the lm() function. Figures were generated using OriginPro 2024 (OriginLab Corp., Northampton, MA, USA) and R software.

## 5. Conclusions

In the sunflower–wheat–maize rotation system of the Hetao Irrigation District, different organic fertilization strategies had significantly different effects on productivity, stability, and nitrogen use efficiency. Among them, CM showed the highest overall performance, followed by CS, whereas CG and CGS did not exhibit clear advantages under the conditions of this study. The CM treatment achieved the highest maize equivalent yield and the lowest coefficient of variation, indicating superior productivity and stability. Meanwhile, CM enhanced crop nitrogen uptake capacity, resulting in increased plant nitrogen accumulation, nitrogen recovery efficiency (NRE), and nitrogen use efficiency (NUE). Therefore, CM can be characterized as a high-yield and stable fertilization strategy, suitable for a wide range of irrigated agricultural systems. The CS treatment also significantly improved system productivity and exhibited strong adaptability to environmental variability. Its advantage in nitrogen use efficiency was mainly reflected in a higher nitrogen internal efficiency (NIE), indicating more efficient conversion of absorbed nitrogen into grain yield. Overall, under irrigated conditions in the Hetao Irrigation District, farmyard manure application and straw incorporation represent two effective fertilization strategies for achieving the coordinated improvement of stable productivity and nitrogen use efficiency in rotation systems.

## Figures and Tables

**Table 1:** Comprehensive benefit score.

Treatment	MEY	CV	*b_i_*	NRE	NIE	NUE	CI
CK	1.00	1.00	1.00	2.01	1.96	1.00	1.33
CM	5.00	5.00	1.50	5.00	2.41	5.00	3.99
CS	2.67	3.59	4.90	1.00	5.00	2.67	3.30
CG	1.49	2.46	5.00	2.98	1.41	1.49	2.47
CGS	1.46	2.30	4.54	3.45	1.00	1.46	2.37

Note: All values are normalized scores on a 1–5 scale; higher scores indicate better performance. MEY, maize equivalent yield; CV, coefficient of variation; *b_i_*, Finlay–Wilkinson regression coefficient; NRE, nitrogen recovery efficiency; NIE, nitrogen internal efficiency; NUE, nitrogen use efficiency; CI, comprehensive index.

**Table 2:** Soil physicochemical properties (0–20 cm) of each treatment prior to the 2023 growing season.

Treatment	SOM (g kg^−1^)	AN (mg kg^−1^)	AP (mg kg^−1^)	AK (mg kg^−1^)
CK	12.6	71.4	25.2	125.7
CM	15.4	91.9	31.4	155.4
CS	14.5	85.5	28.7	143.9
CG	15.1	90.0	29.4	139.7
CGS	15.2	93.3	30.4	149.0

Note: SOM, soil organic matter; AN, alkali-hydrolyzable nitrogen; AP, soil available phosphorus; AK, soil available potassium.

**Table 3:** The amount of chemical fertilizer in different treatments (kg ha^−1^).

Year	Crop	N	P_2_O_5_	K_2_O
2023	Sunflower	270	120	90
2024	Wheat	205	90	60
2025	Maize	240	90	60
2023–2025	Total input	715	300	210

Note: N: nitrogen; P_2_O_5_: phosphorus pentoxide; K_2_O: potassium oxide.

**Table 4:** Nutrient content of organic materials (%).

Type	Farmyard manure	Straw			Green manure
		Sunflower	Wheat	Maize	
C	26.00	50.9	51.3	44.5	61.60
N	0.78	0.69	0.61	0.65	3.80
P	0.15	0.05	0.07	0.05	0.15
K	0.74	1.93	1.14	1.34	2.52

Note: C: carbon; N: nitrogen; P: phosphorus; K: potassium.

## Data Availability

All data generated or used during the study appear in the submitted article.

## Abbreviations

The following abbreviations are used in this manuscript:

CK	Chemical fertilization alone treatment
CM	Farmyard manure application treatment
CS	Straw incorporation treatment
CG	Green manure cultivation treatment
CGS	Green manure incorporation with straw return treatment
PK	Nitrogen-omission treatment
SOM	Soil organic matter
AN	Soil alkali-hydrolyzable nitrogen
AP	Soil available phosphorus
AK	Soil available potassium
MEY	Maize equivalent yield
CV	Coefficient of variation
*b_i_*	Finlay–Wilkinson regression coefficient
NRE	Nitrogen recovery efficiency
NIE	Nitrogen internal efficiency
NUE	Nitrogen use efficiency
CI	Comprehensive index
